# Polypyrrole Percolation Network Gas Sensors: Improved Reproducibility through Conductance Monitoring during Polymer Growth

**DOI:** 10.1021/acsapm.1c01819

**Published:** 2022-03-07

**Authors:** Weishuo Li, Merel J. Lefferts, Ben I. Armitage, Krishnan Murugappan, Martin R. Castell

**Affiliations:** Department of Materials, University of Oxford, Parks Road, Oxford OX1 3PH, U.K.

**Keywords:** conducting polymer, gas sensor, percolation network, in situ electrochemical conductance, electrochemical polymerization, device-to-device reproducibility

## Abstract

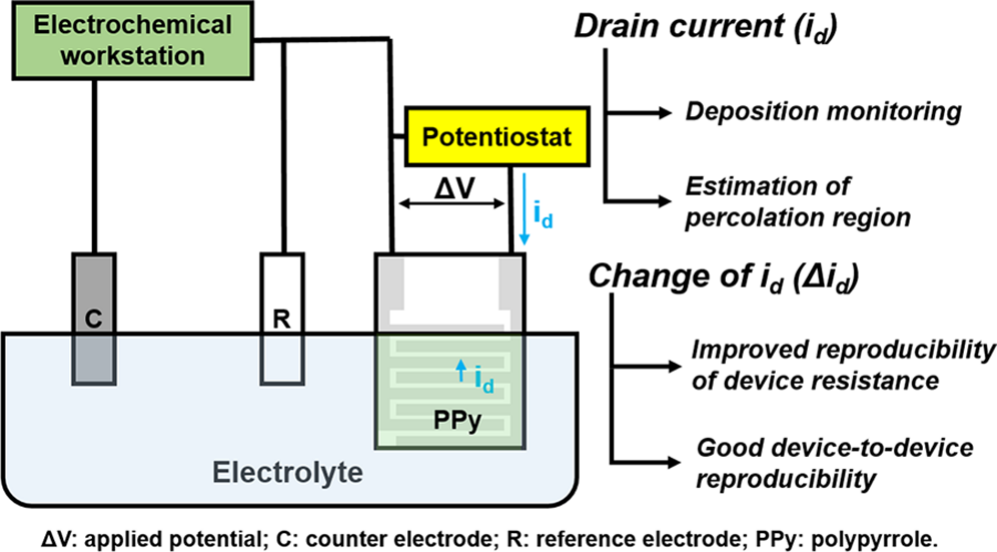

Conducting-polymer-based electrical percolation networks are promising materials for use in high-sensitivity chemiresistive devices. An ongoing challenge is to create percolation networks that have consistent properties, so that devices based on these materials do not have to be individually calibrated. Here, an in situ conductance technique is used during the electrochemical growth of polypyrrole (PPy) percolation networks. The drain current (*i*_d_) across the interdigitated electrodes (IDEs) is a measure of the conductance of the PPy network during electrochemical polymerization. The *i*_d_ curve is used to determine the percolation region. To improve the reproducibility of PPy percolation networks, an in situ conductance monitoring method based on the value of *i*_d_ is used. A set of optimal ammonia gas percolation sensors was created using this method with an average sensitivity of Δ*R*/*R*_0_ × 100% ppm^–1^ = 11.3 ± 1.2% ppm^–1^ and an average limit of detection of 15.0 ± 3.6 ppb.

## 1 Introduction

Conducting polymers (CPs) were first discovered in the 1970s,^1−3^ and due to their unique properties, they are now used in a wide range of applications.^4−9^ Polymer-based chemiresistors are regarded as one of the simplest ways to achieve chemical sensing.^10^ To form CP-based chemiresistors, CPs are deposited as a sensing layer, and interactions between the CPs and the analyte molecules cause changes in the electrical properties of the CP layer that can be easily monitored.

Sensitivity is one of the most significant parameters for the sensing performance of chemiresistors. Increasing the surface-to-volume ratios of sensing layers has to date been considered as the most effective way to improve their sensitivity. To achieve this, CPs have been fabricated through complex processes into different forms, including ultrathin films, porous thin films, and finely designed nanostructures.^9,11^ However, it is still challenging to achieve ultrahigh sensitivity at low analyte concentrations using straightforward processing methods.

**Figure 1:**
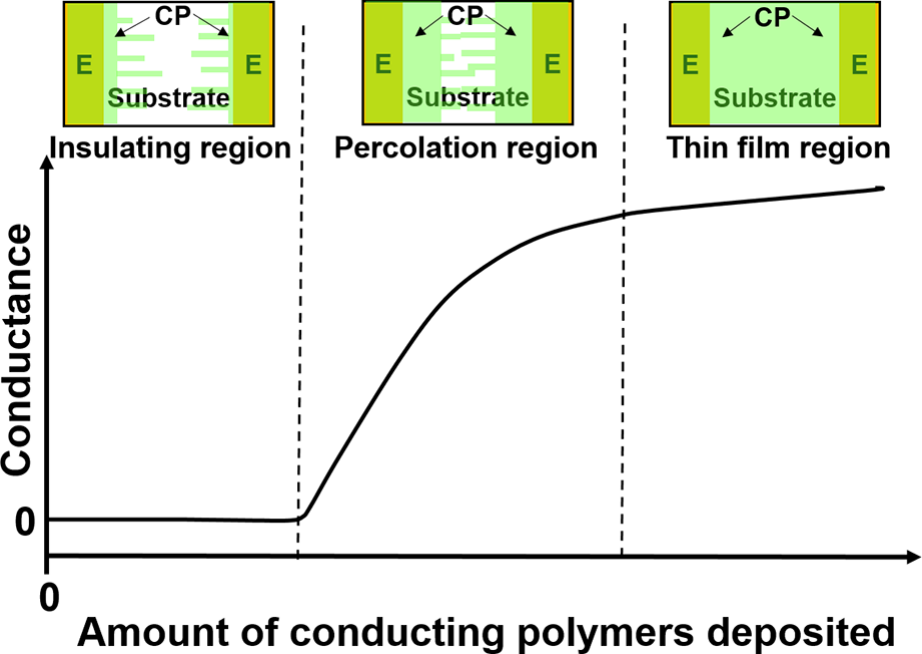
Electrical conductance between two electrodes as a function of the amount of conducting polymer deposited between the electrodes. The three main regions are the insulating, percolation, and dense thin-film regions (CP: conducting polymer; E: electrode).

Recently, it was shown that electrical percolation materials have significantly increased sensitivity over their dense thin-film counterparts.^12−14^ In our context, electrical percolation is identified by the sharp increase in conductance between two electrodes when fabricating a chemiresistor via electropolymerization (Figure 1). Initially, CPs only grow on the surface of the electrodes, resulting in no conductance between the electrodes. This is known as the insulating region. As growth proceeds, a small number of CPs start to expand across the substrate and eventually form an electrical bridge between the electrodes. A sharp increase in the conductance is observed, indicating that the percolation region has been reached. Lastly, the dense thin-film region is reached as more CPs are deposited. The conductance increases slowly in this region as the film thickness becomes the major factor controlling the film conductance. Compared to chemiresistive sensors in the thin-film region, chemiresistive sensors in the percolation region are more sensitive because a small number of interactions between the CPs and the analyte gas will lead to a large resistance change. The conductivity of the whole percolation pathway can be disrupted by the analyte on polymer connections, while the analyte will only affect the local surface conductivity of the dense thin film. Our previous work has shown that electropolymerization can be used to fabricate chemiresistors based on polypyrrole (PPy) and poly(3,4-ethylenedioxythio-phene) (PEDOT) percolation networks and that the sensitivities and limits of detection of the resulting percolation networks to ammonia, nitrogen dioxide, and ammonium nitrate/fuel oil are significantly improved compared to more traditional thin-film chemiresistive sensors.^15−19^

Challenges remain when creating percolation sensors on a routine basis. Percolation networks of CPs are prepared by electropolymerization, and the percolation region can be determined after plotting the electrical conductance obtained after different numbers of deposition cycles or times. This method does not allow for continuous monitoring of the conductance of the evolving CP network, and because of the inherent randomness of network connections it is challenging to achieve reproducible results, even for CP layers produced with identical procedures. In the percolation region, small variations in the network have a large effect on the conductance because of the random nature of the polymer growth,^20,21^ thus variations in conductance between samples can be up to an order of magnitude. As the sensitivity and limit of detection are related to the initial conductance of the chemiresistor,^15^ the variations in conductance observed in the percolation region cause variations in sensing performance, leading to unsatisfactory device-to-device reproducibility. Few studies have discussed this reproducibility issue for percolation sensors, revealing a significant gap in the understanding of how to circumvent this problem.

**Figure 2:**
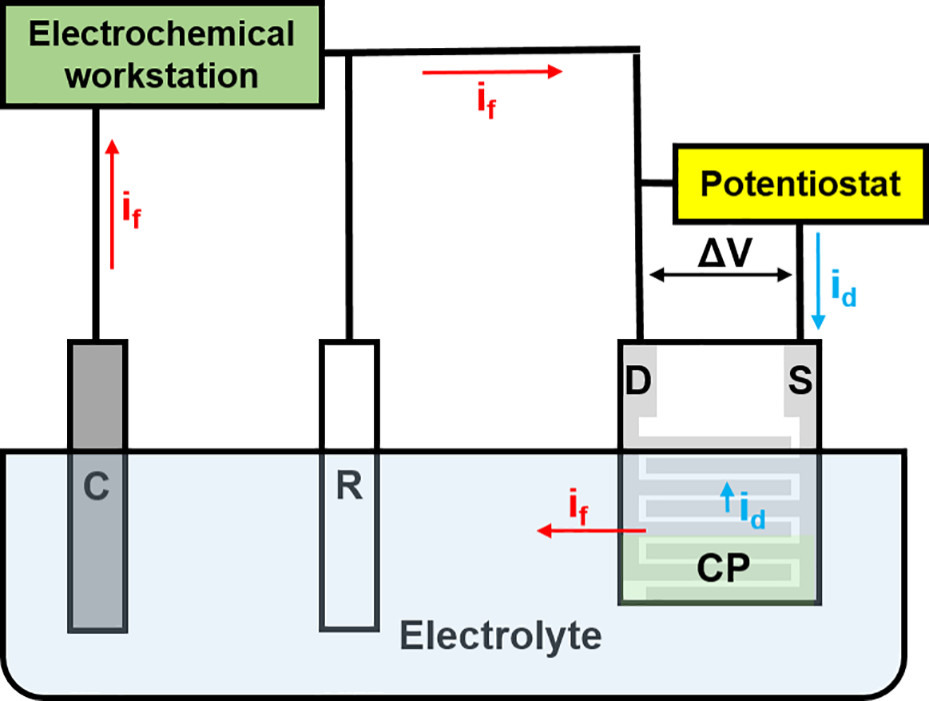
Schematic diagram of the electric circuit for in situ conductance monitoring. C: counter electrode; R: reference electrode; S: source; D: drain; CP: conducting polymer deposition region; *i*_f_: faradaic current; *i*_d_: drain current that flows across the interdigitated electrodes due to Δ*V*.

In situ conductance measurement is an ideal way to gain real-time insight into the electrical properties of CP films during electrochemical growth,^22^ enabling control of the electropolymerization process in the percolation region. Using a three-electrode setup, a potentiostat is introduced between the IDEs (Figure 2). The electrochemical workstation provides the faradaic current (*i*_f_) and controls the deposition process. The drain current (*i*_d_) induced by the applied potential (Δ*V*) is recorded by the potentiostat, reflecting the resistance across the IDEs in real time. A similar technique has been reported for the fabrication of chemiresistors based on a CP film^23^ but has to date not been applied to percolation networks.

Herein, in situ conductance monitoring is used to investigate PPy-based percolation networks, the reproducibility of their initial resistances, and the device-to-device reproducibility of percolation sensors. The *i*_d_ curves of PPy recorded during chronoamperometric electropolymerization are analyzed, and their reliability to effectively estimate the percolation region is discussed. Next, an in situ conductance monitoring method, based on the measurement of *i*_d_, is introduced to control the deposition process. Using this method, improved reproducibility of the initial resistances of PPy networks is demonstrated, and the device-to-device reproducibility for percolation sensors is investigated.

## 2 Experimental Section

### 2.1 Electrochemical Experiments

All the chemicals for electrochemical deposition were purchased from Sigma-Aldrich (UK). Pt interdigitated electrodes (IDEs) were purchased from Micrux (Spain). Each IDE consists of 180 pairs of 5 μm wide Pt electrodes separated by a gap of 5 μm on an insulating glass substrate. Prior to the electrochemical experiments, the IDEs were cleaned with concentrated nitric acid (HNO_3_, 90%) and sonicated in methanol (CH_3_OH, 99.9%), ethanol (C_2_H_5_OH, 99.8%), and acetone (C_3_H_6_O, 99.8%) for 10 min each.

Electrochemical experiments were performed using a PGSTAT204 Autolab electrochemical workstation (Eco Chemie, Netherlands) interfaced to a PC with NOVA version 1.11 software. The three-electrode cell used a Pt coil (BASi, USA) as the counter electrode, an Ag/AgCl (CH Instruments, USA) as the reference electrode, and a Pt IDE as the working electrode. A B2900A potentiostat (Keysight, UK) was used to achieve the setup for in situ conductance monitoring. PPy was synthesized by chronoamperometry at 1.0 V from a supporting electrolyte of 50 mM pyrrole (Py, 98%) and 0.1 M lithium perchlorate (LiClO_4_, 95%) in acetonitrile (CH_3_CN, 99%). During deposition, the *i*_d_ current was recorded as a function of time via a PC equipped with Benchvue software interfaced to the potentiostat. Samples were prepared with applied potentials (Δ*V*) from 20 to 80 mV. Samples with various conductance values were prepared by halting electropolymerization at different deposition times or values of *i*_d_. After deposition, samples were washed with CH_3_CN and dried in air for 10 min. This washing and drying process was repeated after each sample was p-doped at 1.0 V for 60 s in an electrolyte solution of 0.1 M LiClO_4_ in CH_3_CN.

### 2.2 Sensing Experiments

**Figure 3:**
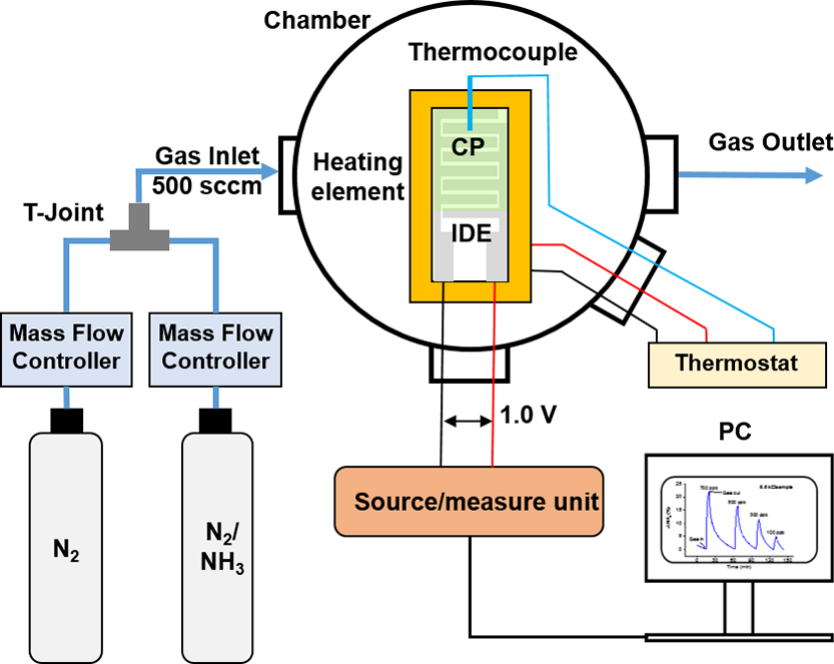
Schematic layout of the experimental setup for ammonia sensing measurements.

Sensing experiments were carried out in a custom-made sensing chamber at atmospheric pressure (Figure 3). Ammonia gas (10 ppm, nitrogen fill) and nitrogen gas (for further dilution of the ammonia) for sensing tests were purchased from BOC Gases UK. The flow rate from each gas cylinder was controlled by a mass flow controller (Alicat). Gases were mixed at a T-joint before entering the gas inlet of the chamber, and a constant total flow rate was maintained at 500 standard cubic centimeters per minute (sccm) throughout the experiments. The concentration of ammonia gas was determined by the relative flow rates of the two mass flow controllers. Each sensor was placed on a sample stage equipped with a heating element, and a thermocouple was fixed near its sensing layer. The temperature around the sensor was maintained at 27 °C via a thermostat. The sensor in the chamber was connected to a B2900A source/measure unit (Keysight, UK). The chamber was first purged with nitrogen gas for 30 min to remove impurities from the chamber and the sensing layer. Then a dc potential of 1.0 V was applied across the two interdigitated electrodes of the sensor, and the current was monitored on a PC equipped with Benchvue software interfaced to the source/measure unit. Once a stable baseline of current was reached, the sensor was exposed to concentrations of ammonia gas from 1 to 4 parts per million (ppm) for 1 min each. After each exposure, the sensor was purged with nitrogen until the current returned to the baseline before the next exposure.

## 3 Results and Discussion

### 3.1 Drain Current Curves

**Figure 4:**
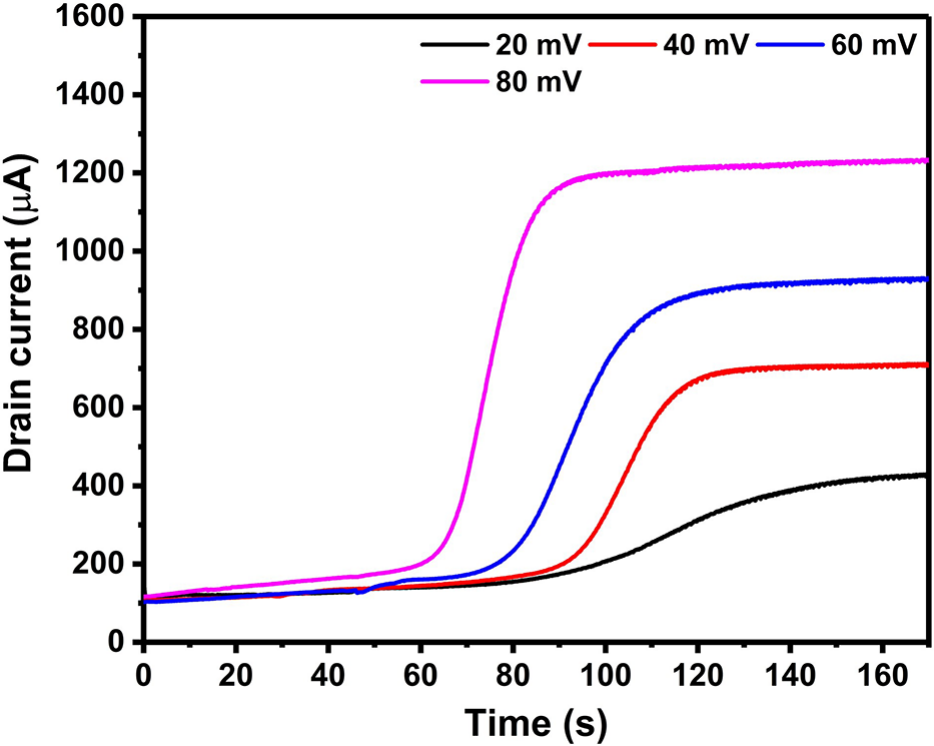
Measured *i*_d_ current curves during chronoamperometric polymer growth with Δ*V* of 20, 40, 60, and 80 mV. The curves show an earlier onset of the current increase and a higher final *i*_d_ for higher Δ*V*s.

PPy networks were prepared via chronoamperometry with Δ*V* of 20, 40, 60, and 80 mV. The *i*_d_ current during deposition was measured to study the trend of conductance between the IDEs in the electrolyte. All the *i*_d_ curves exhibit similar trends from baselines to plateaus (Figure 4). Initially, PPy nucleates and grows on the Pt IDE fingers; this is the baseline for each *i*_d_ curve. The initial *i*_d_ is not zero as a part of *i*_f_ flows through the potentiostat. Once the fingers are fully covered, PPy starts to grow into the insulating gaps between IDE fingers. As deposition proceeds, *i*_d_ increases sharply owing to the formation of PPy bridges between IDE fingers. A higher Δ*V* results in a shorter time to reach the onset of the sharp increase in gradient because the Δ*V* is added onto the deposition potential for one side of electrodes and thus speeds up the deposition on that side.^22^ The *i*_d_ current keeps increasing until a plateau is reached, indicating the fingers are fully connected and a PPy dense thin film has formed. The height of the plateau depends on Δ*V* owing to Ohm’s law. These *i*_d_ curves are consistent with reported trends describing the conductance changes from the insulating region to the thin-film region.^15,16,19^ This suggests that our in situ method enables continuous monitoring of the growth of PPy between the IDEs.

A suitable single value of Δ*V* needs to be decided upon before further studies are carried out on the reliability of the *i*_d_ curve and the reproducibility of the percolation networks. Although a very low *i*_d_ induced by a very low Δ*V* can avoid the overoxidation of the CP on one side of the electrode, it will result in relatively higher noise. For real-time monitoring, the change of the display range for *i*_d_ should also be taken into consideration. For instance, 80 mV is not suitable since the corresponding *i*_d_ increases over 1000 μA after 80 s, and the display unit of *i*_d_ has to change from μA to mA. Considering these factors, both 60 mV and 40 mV are suitable; however, we chose 60 mV to obtain a better signal-to-noise ratio. Using a Δ*V* of 60 mV, the *i*_d_ current starts to increase sharply after 70 s, and the *i*_d_ plateau is reached after 120 s (Figure 4).

### 3.2 In Situ Time-Controlled Method

**Figure 5:**
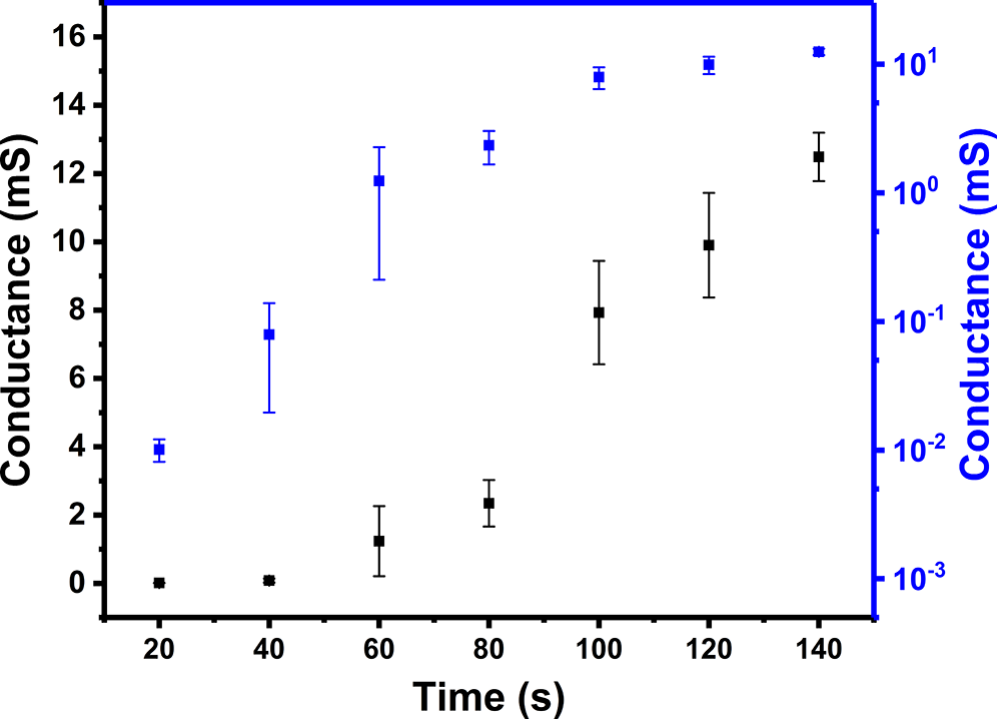
Plot of conductance vs deposition time (in black) and its semilogarithmic plot (in blue) for the deposition of PPy on Pt IDEs using an in situ time-controlled method at 1.0 V and a Δ*V* of 60 mV for deposition times of 20–140 s. All the error bars are obtained from the standard deviation of the conductance values of four separate samples.

A time-controlled preparation method was used to study the reliability of the *i*_d_ curve. PPy was grown by chronoamperometry at 1.0 V using Δ*V* = 60 mV. Four samples were obtained at each deposition time ranging between 20 and 140 s. Their resistances were recorded after the post-treatment processes including doping, washing, and drying (Figure 5). The semilogarithmic plot in blue is used to determine the boundary between the percolation and the thin-film regions. The conductance increases sharply at first, showing the percolation behavior. Then, it starts to increase slowly at 100 s within the same order of magnitude, indicating that percolation ends after around 100 s. Some error bars in conductance for the percolation region are quite large, covering an order of magnitude. This is in agreement with previous work based on the time-controlled method.^15,19^

**Figure 6:**
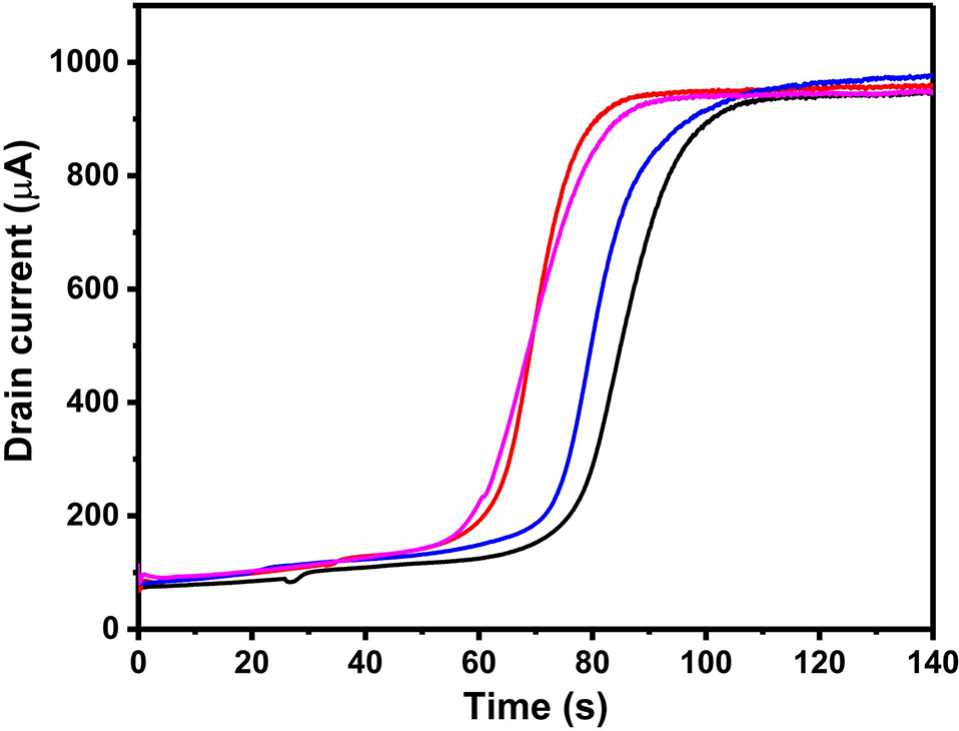
Drain current (*i*_d_) curves for 4 independent samples during chronoamperometric growth using in situ conductance measurement with Δ*V* = 60 mV. The onset of the significant current increase varies from around 55 to 70 s.

The normal plot in black determined using this time-controlled method (Figure 5) is similar to the percolation curve of the *i*_d_ current using Δ*V* = 60 mV (Figure 4). A possible reason for any difference could be that the sample for the *i*_d_ curve under 60 mV in Figure 4 only represents one possibility, whereas the data in Figure 5 represents the average of 4 independent samples. To demonstrate this, *i*_d_ curves were recorded for Δ*V* = 60 mV for 4 independent samples (Figure 6). For these 4 measurements, the onset of the significant current increase varies from 55 to 70 s, which is similar to the results at 60 s obtained with the time-controlled method. The variability in the onset of the current increase is attributed to the random growth of PPy. Although the difference in conductance and the variability of the *i*_d_ curve are unavoidable, the *i*_d_ curve is reliable enough to provide a reference range to estimate the percolation behavior for an unknown system, improving the accuracy and efficiency for determining the percolation region in practical experiments. The variability of the *i*_d_ curves in Figure 6 also shows the drawback of the time-controlled method, requiring an alternative method to improve the reproducibility of the initial conductance of the CP networks.

### 3.3 In Situ Current-Controlled Method

**Figure 7:**
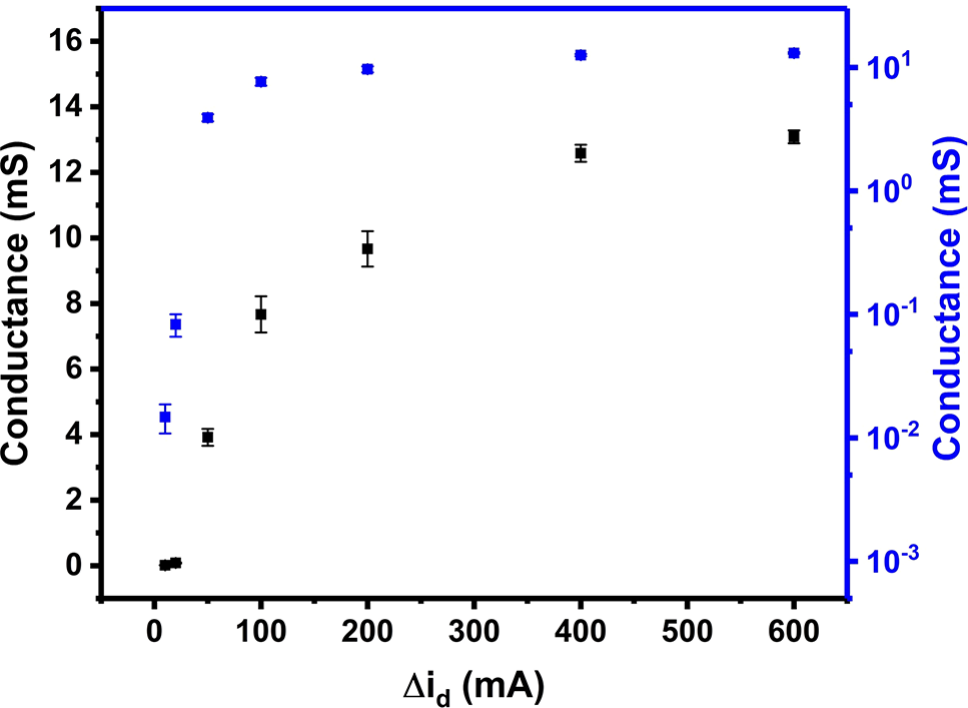
Plot of conductance vs Δ*i*_d_ (in black) and its semilogarithmic plot (in blue) for the growth of PPy on Pt IDEs. All the error bars are obtained from the standard deviation of the conductance values of four separate samples.

To improve the reproducibility of our percolation networks, a current-controlled method was designed, using the difference of *i*_d_ between the baseline and the value during growth (Δ*i*_d_). PPy was grown via chronoamperometry at 1.0 V and Δ*V* = 60 mV. Instead of halting the deposition after a predetermined time, as in the time-controlled method, samples were prepared by stopping the growth when Δ*i*_d_ reached a predetermined value between 10 and 600 μA. Then, three more repeat samples were created for each value of Δ*i*_d_. Their resistances were recorded following the post-treatment processes including doping, washing, and drying (Figure 7). Small measurable Δ*i*_d_ values indicate that the percolation threshold has been reached where a small number of polymer bridges have been created between the electrodes. However, for practical purposes, sensors with very low conductance values are unsuitable because their signal-to-noise ratios tend to be very high.

Variations in conductance remain in the data in Figure 7 because the conductance is affected by post-treatment, for example when the sample is washed or doped. Compared to the time-controlled method (Figure 5), the current-controlled method shows smaller error bars, significantly improving the reproducibility of the conductance of the CP networks, especially in the percolation region. These differences are more discernible in the semilogarithmic plots. These smaller variations in conductance from the current-controlled method are attributed to a more direct way of monitoring the electrical properties of PPy during growth instead of using the deposition time to indirectly represent the amount of PPy obtained after growth.

It should be noted that the reproducibility of the conductance plays a significant role in studies of the sensing performance of the CP networks. Previous work has shown that the initial conductance affects the sensitivity.^15,16^ Compared to dense thin-film chemiresistive sensors with higher initial conductances, percolation chemiresitive sensors with lower initial conductances exhibit higher sensitivities. Furthermore, networks with similar values of initial conductance will exhibit similar signal and noise levels during sensor testing, resulting in similar limits of detection. Although percolation is a promising approach to easily obtain improved sensor performances, challenges for further studies and real-life applications remain if there is no effective way to produce percolation sensors with reliable and repeatable properties. The in situ current-controlled method makes it possible to overcome this by improving the reproducibility of the initial conductance of the CP network.

### 3.4 Sensor Performance

**Figure 8:**
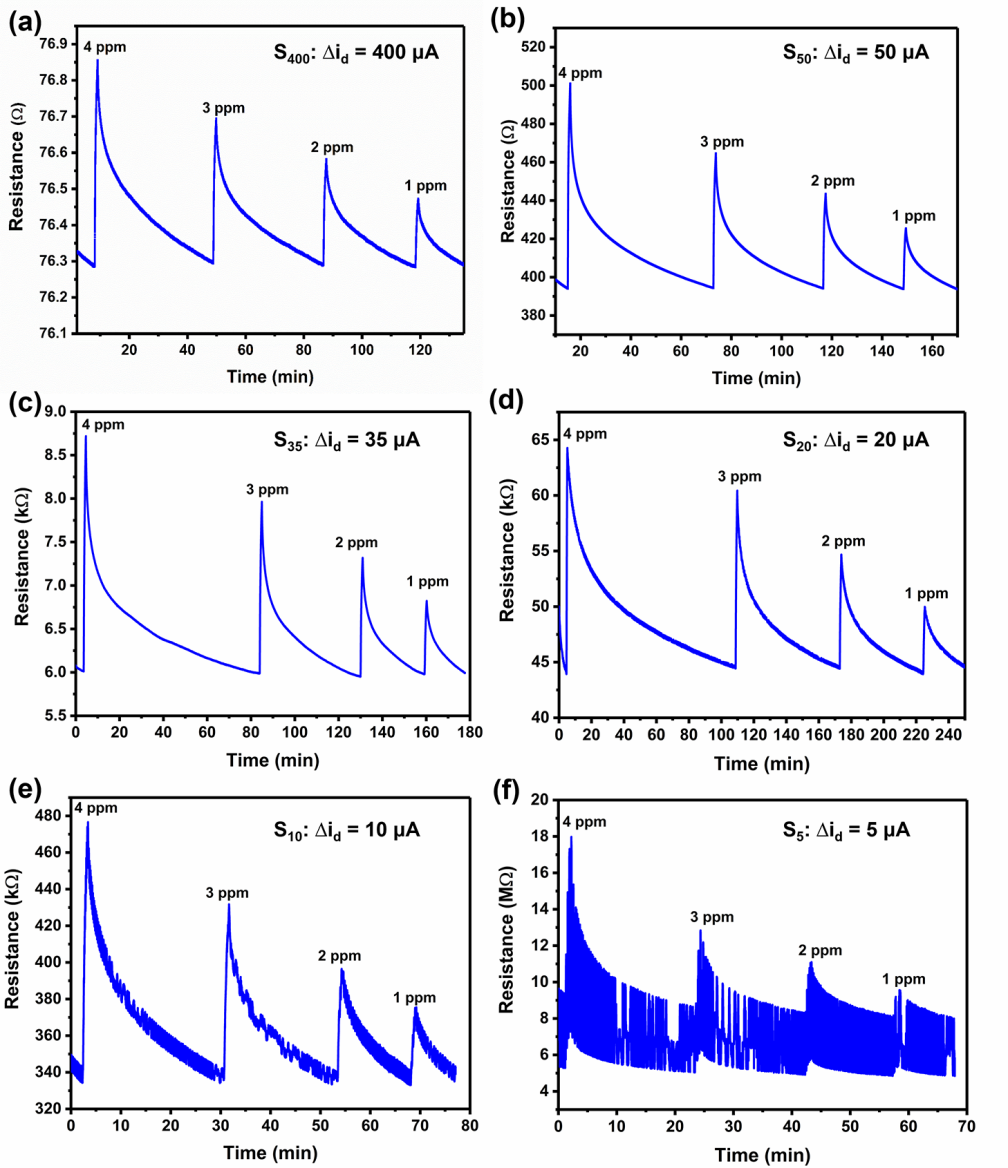
Sensing responses to 4, 3, 2, and 1 ppm ammonia in N_2_ for PPy-based chemiresistors obtained by the in situ current-controlled method at Δ*i*_d_ values of (a) 400, (b) 50, (c) 35, (d) 20, (e) 10, and (f) 5 μA. Fresh ammonia exposures were only conducted once the sensor had recovered to the baseline. The current value in the upper right corner of each figure represents the Δ*i*_d_ value used to fabricate the sensor.

The in situ current-controlled method was used to prepare PPy-based chemiresistors for sensing experiments. Sensors called S_400_, S_50_, S_35_, S_20_, S_10_, and S_5_ were fabricated by stopping growth at Δ*i*_d_ values of 400, 50, 35, 20, 10, and 5 μA, respectively. Halting PPy growth with a smaller value of Δ*i*_d_ results in sensors with lower conduction. The sensors were placed in the sensor testing chamber under N_2_ flow, and 1.0 V was applied across the IDEs. The initial resistance of each sensor is defined as the resistance obtained after the baseline is reached. Sensors were then exposed to 1–4 ppm ammonia gas, and the sensor response was recorded (Figure 8). As expected, the resistances increase during the exposure to ammonia gas because electron-donating ammonia molecules reduce the number of hole charge carriers in p-doped PPy. The resistance changes were reversible, returning to the baseline after exposure. Sensors with higher initial resistances displayed higher levels of noise. Among these sensors, S_5_ with the largest resistance was not used for further experiments since it was difficult to distinguish signal from noise.

**Figure 9:**
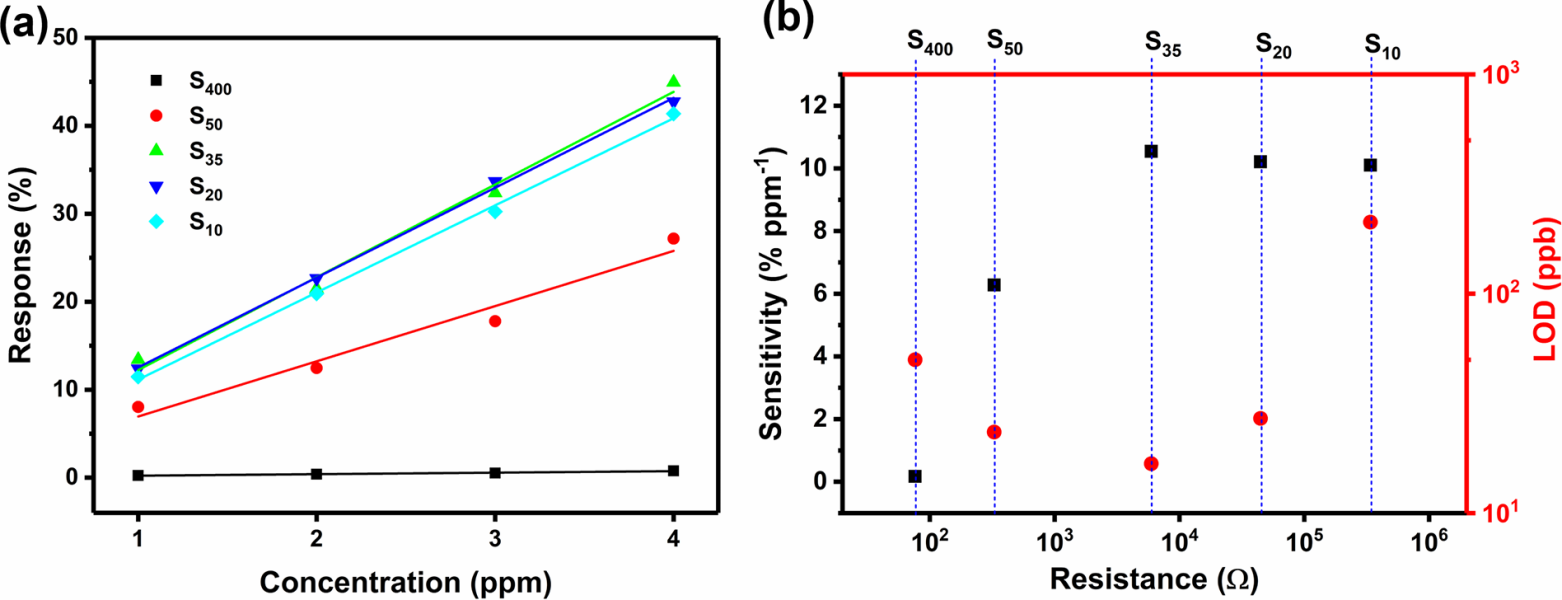
(a) Sensing responses, defined as Δ*R*/*R*_0_ × 100%, as a function of ammonia concentration for sensors S_400_, S_50_, S_35_, S_20_, and S_10_. (b) Values of sensitivity (in black) and limits of detection (in red) for the five PPy-based sensors investigated.

To achieve the optimal sensor, the sensing responses of S_400_, S_50_, S_35_, S_20_, and S_10_ were obtained by calculating the percentage changes to the initial resistances (Δ*R*/*R*_0_ × 100%). For each sensor, a linear relationship was observed between the sensing response and the concentration of ammonia (Figure 9a). The sensitivity is defined as the slope of the linear fit in Figure 9a, and the limit of detection (LOD) is defined as three times the standard deviation of the baseline noise level (σ) divided by the sensitivity. Figure 9b shows that S_400_, obtained in the thin-film region, exhibits the lowest sensitivity. As the initial resistance increases, the sensitivity also increases and becomes stable above 10% ppm^–1^, where percolation networks determine the sensitivity instead of dense thin films. In the percolation region, interactions between the percolation network and an analyte molecule have a larger effect on the resistance of the networks, resulting in a significantly increased sensitivity compared to thin-film-based sensors. Although S_20_ and S_10_ have high sensitivities, they also have higher LODs because their noise levels are relatively large compared to their sensitivity. As a result, as the starting resistance of the sensor increases, the LOD first decreases and then increases owing to the competitive relationship between sensitivity and baseline noise. Therefore, S_35_ is regarded as the optimal sensor with a sensitivity of 10.5% ppm^–1^ and an LOD of 17 ppb. This is in agreement with previous work where the percolation region was found at 1–10 kΩ for sensors created with the time-controlled method.^15^ This demonstrates that the in situ current-controlled method, while increasing control over sensor preparation by halting polymer growth at a predetermined Δ*i*_d_ instead of deposition time, results in the same resistance range for optimal sensors.

**Figure 10:**
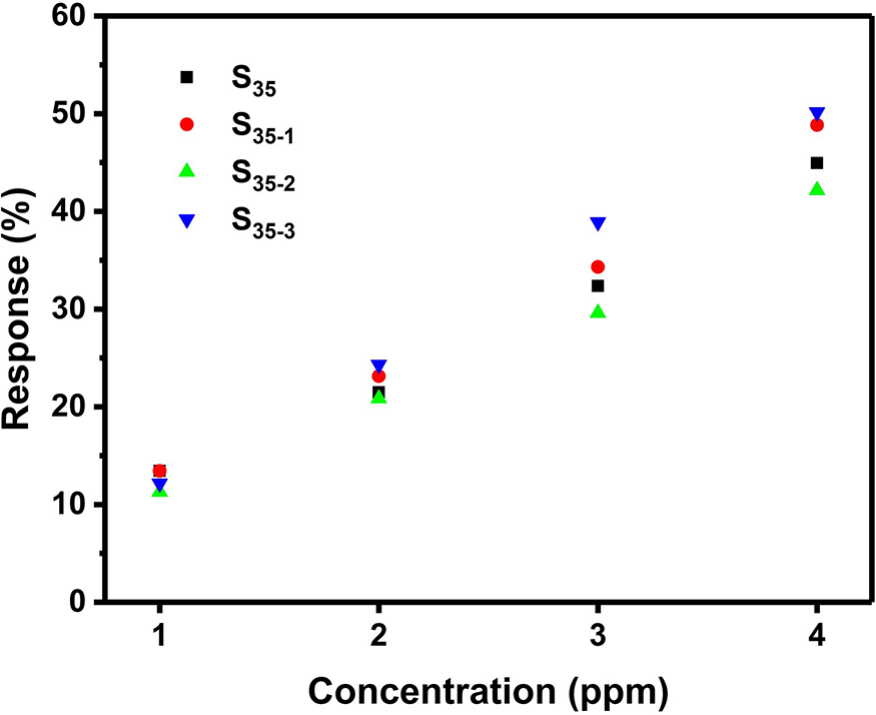
Sensing responses as a function of ammonia concentration for S_35_, S_35-1_, S_35-2_, and S_35-3_.

Sensor S_35_ was reproduced 3 times to study the device-to-device reproducibility. Sensors S_35-1_, S_35-2_, and S_35-3_ were prepared and tested under the same conditions as sensor S_35_. The sensor responses to 1–4 ppm ammonia obtained for S_35-1_, S_35-2_, and S_35-3_ were similar to those of S_35_ (Figure 10). In the literature, sensing responses at various concentrations were used to evaluate the device-to-device reproducibility for CP-based chemiresistors.^24−27^ The coefficient of variation (CV), defined as the mean divided by the standard deviation, for responses with desirable reproducibility varies from 0.05 to 0.10. To evaluate the device-to-device reproducibility of our percolation sensors, the CV value for the responses at each concentration in Figure 10 was calculated. The average of these CV values is 0.0870. This indicates that our percolation sensors also exhibit a desirable device-to-device reproducibility of sensing response, achieved with the in situ current-controlled method.

Sensitivities and LODs were calculated for all S_35-*x*_ sensors. As shown in Table 1, S_35_ and its reproduced sensors exhibit similar initial resistances, within the same order of magnitude. The variation in their initial resistances is attributed to the random effects from postgrowth doping as well as the stabilization before the baseline is reached. The CV for their resistances is smaller than the value of 0.2058 for samples obtained at 20 μA in Figure 7. This indicates that the variation in resistance at 35 μA is further reduced. Furthermore, all four sensors have similar sensitivities. This is likely because their similarity in initial resistance results in a similar number of conducting pathways in their percolation networks, resulting in similar sensing behavior. The average sensitivity for this series of optimal sensors is 11.3 ± 1.2% ppm^–1^. The CV value for the standard deviation in the baseline of the sensing response (σ) is similar to that for initial resistance because σ is related to the initial resistance. The difference between the CV for σ and the CV for resistance may be attributed to other factors that could affect the σ, such as the testing setup or environment. As for the LOD, it depends on a comprehensive contribution from sensitivity and σ, leading to a relatively larger CV value of 0.2372 for all four percolation sensors. Their average LOD is 15.0 ± 3.6 ppb. This demonstrates that good reproducibility of CP percolation network sensors can be achieved with the in situ current-controlled method.

**Table 1:** Summary of PPy-Based Percolation Sensors Prepared at a Δ*i*_d_ of 35 μA

sensor	initial resistance (Ω)	sensitivity (% ppm^–1^)	σ^a^ (%)	LOD (ppb)
S_35_	5949	10.5	0.0589	17
S_35-1_	4480	11.7	0.0684	18
S_35-2_	4882	10.1	0.0508	15
S_35-3_	3895	12.8	0.0425	10
CV^b^	0.1803	0.1085	0.2009	0.2372

a The standard deviation of the baseline in sensing response.

b Coefficient of variation (CV) = mean/standard deviation.

## 4 Conclusion

An in situ conductance technique was used to study PPy-based percolation networks, the reproducibility of their initial resistances, and the device-to-device reproducibility of chemiresistive gas sensors based on such percolation networks. During deposition the *i*_d_ curve reflects the conductance of the PPy networks in real time, spanning from the insulating region to the thin-film region. Compared to the conductance plot obtained by the time-controlled method, it is demonstrated that the *i*_d_ curve can be used to reliably estimate the percolation region and improve the reproducibility of the initial conductance of the percolation networks. Using the current-controlled method, a series of optimal percolation sensors (S_35_) with a sensitivity of 11.3 ± 1.2% ppm^–1^ and an LOD of 15.0 ± 3.6 ppb to ammonia gas were created. Our conductance-controlled method enables opportunities for larger scale sensor fabrication with relevance to commercialization of percolation network sensors.
